# Orthodontic Bracket Removal and Enamel Roughness: Comparing the Effects of Sapphire and Metallic Brackets in an In Vitro Study

**DOI:** 10.3390/bioengineering12101041

**Published:** 2025-09-28

**Authors:** Cosmin Bogdan Licsăndroiu, Mihaela Jana Țuculină, Adelina Smaranda Bugălă, Petre Costin Mărășescu, Felicia Ileana Mărășescu, Andreea Gabriela Nicola, Cristian Niky Cumpătă, Cosmin Mihai Mirițoiu, Ovidiu Ioan Gheorghe, Maria Cristina Bezna, Elena Verona Licsăndroiu, Ionela Teodora Dascălu

**Affiliations:** 1 Doctoral School, University of Medicine and Pharmacy of Craiova, 200349 Craiova, Romania; licsandroiubogdan@yahoo.com (C.B.L.); ovis45@yahoo.com (O.I.G.); 2 Department of Endodontics, Faculty of Dental Medicine, University of Medicine and Pharmacy of Craiova, 200349 Craiova, Romania; mtuculina@yahoo.com (M.J.Ț.); preda.smaranda@yahoo.com (A.S.B.); 3 Department of Dental Prothesis Technology, Faculty of Dental Medicine, University of Medicine and Pharmacy of Craiova, 200349 Craiova, Romania; 4 Department of Orthodontics, Faculty of Dental Medicine, University of Medicine and Pharmacy of Craiova, 200349 Craiova, Romania; ciuca_felicia@yahoo.com (F.I.M.); marceldascalu@yahoo.com (I.T.D.); 5 Department of Preventive Dentistry and Oral Health, Faculty of Dental Medicine, University of Medicine and Pharmacy of Craiova, 200349 Craiova, Romania; andeea_anghel@yahoo.com; 6 Department of Oromaxillofacial Surgery, Faculty of Dental Medicine, University Titu Maiorescu of Bucharest, 031593 Bucharest, Romania; 7 Department of Applied Mechanics and Civil Constructions, Faculty of Mechanics, University of Craiova, 200512 Craiova, Romania; miritoiu_cosmin@yahoo.com; 8 Department of Pathophysiology, Faculty of Dentistry, University of Medicine and Pharmacy of Craiova, 200349 Craiova, Romania; bezna.mariacristina@gmail.com; 9 Ciso Medical Bucharest, B-vd Burebista, No.1, 031106 Bucharest, Romania; licsandroiuverona@gmail.com

**Keywords:** enamel roughness, orthodontic debonding, metallic brackets, sapphire brackets, remineralization, surface analysis, SEM, profilometry

## Abstract

**Background:** Enamel surface roughness after bracket debonding is an important issue due to its impact on plaque accumulation and the potential development of carious lesions. This in vitro study aimed to assess enamel roughness after the removal of metallic and sapphire brackets and the effect of a remineralization treatment. **Methods:** Two hundred extracted human permanent teeth with healthy enamel were randomly distributed into two groups (*n* = 100) and bonded with either metallic or sapphire brackets using the same adhesive (3M™ Transbond™ XT (St. Paul, MN, USA), Minnesota Mining and Manufacturing Company, MN, USA). The enamel surface roughness was measured before bonding, after debonding, and after remineralization using SEM and a TR200 roughness (SaluTron GmbH, Frechen, Germany) tester. The parameter Ra was used to quantify the surface roughness. One-way ANOVA, the normality test, variance homogeneity, and the Bonferroni post hoc test were used to analyze the data. **Results:** Debonding significantly increased the enamel surface roughness in both groups. The sapphire bracket group presented significantly higher mean Ra values post debonding (4.14 ± 0.36 µm) compared to the metallic group (2.56 ± 0.52 µm). Remineralization led to a decrease in surface roughness in both groups, though not to baseline levels. The changes were statistically significant (*p* < 0.01), with a power of the test of 1.0. **Conclusions:** The bracket material significantly affects enamel surface roughness after orthodontic debonding. Sapphire brackets produced greater surface irregularities than metallic ones. Remineralization partially reduced roughness in both groups, with the final values in the metallic group being closer to baseline levels. Crucially, these values remained far above the clinical threshold for plaque retention, highlighting the need for improved debonding techniques.

## 1. Introduction

Tooth enamel is the hardest and most highly mineralized material in the human body, composed of more than 95% inorganic material, primarily hydroxyapatite crystals organized in a hierarchical prismatic structure. This arrangement confers resistance to mechanical wear and acid erosion. However, as a non-vital and acellular tissue, enamel lacks regenerative capacity. Remineralization is only possible during its early stages and depends on oral pH dynamics and the presence of specific ions or agents [1,2]. Recent advances in imaging techniques, such as synchrotron micro-XRF (Bruker AXS, Karlsruhe, Germany) and high-resolution microscopy, have further highlighted the vulnerability of enamel microstructure and the importance of preserving enamel integrity during dental interventions [3,4].

Enamel thickness varies across the tooth surface, reaching up to 2.5 mm at the cusp tips and incisal edges, and thinning near the cemento-enamel junction [4,5,6,7,8,9,10,11]. In areas such as cervical margins or occlusal pits, aprismatic enamel, characterized by higher mineral and fluoride content, provides additional resistance to acidic challenges [3,4,5,6]. Despite this structural resilience, orthodontic procedures such as bracket bonding, mechanical stripping, and adhesive removal can cause irreversible enamel alterations, including increased surface roughness, microcracks, and demineralization. Such alterations can promote plaque retention and surface discoloration, thereby increasing the risk of caries [5,12,13,14,15,16,17].

In particular, enamel stripping—used to manage arch length discrepancies—results in persistent roughness even after polishing. While several studies report a correlation between stripping and increased plaque accumulation, the relationship between surface roughness and caries remains inconclusive [12,13,14,15,16,17]. Other common procedures, such as acid etching with 37% orthophosphoric gel, or abrasive pre-bonding techniques, also contribute to surface degradation [6,7].

The characteristics of the orthodontic brackets used may also influence enamel surface condition. Sapphire brackets (MBT 5-5UP/LW-HUBIT) are made of monocrystalline material and are valued for their high translucency and biocompatibility. Their base design includes a textured surface to enhance adhesion and facilitate clean debonding, thereby reducing the risk of enamel damage. Their curved shape allows for uniform force distribution and improved adaptation to the vestibular tooth surface. By contrast, metallic brackets are designed for maximum mechanical efficiency, with an extended base surface that enhances adhesive retention. Their anatomical design promotes secure fit and improved patient comfort during treatment. Both bracket types incorporate structural elements such as wing spacing for elastic anchorage and low-friction contours to improve rotational control and reduce treatment time [12,13,14,15].

Residual adhesive left on the enamel surface following bracket debonding is another factor which contributes to increased surface roughness and plaque retention [8,9,10,11]. Resin-modified glass ionomer cements have been proposed as alternatives for bracket bonding due to their potential to minimize enamel alterations and reduce residual material [7,11,18,19]. Increased roughness is typically regarded as undesirable, as it indicates surface irregularities that may affect enamel health and esthetics [20]. Quantitative roughness assessment is based on parameters defined by SR EN ISO 21920/2-2022 [21].

From a bioengineering perspective, the bonded orthodontic bracket represents a complex multi-material interface, comprising the biological enamel substrate, a polymeric adhesive, and an engineered bracket material. The clinical success of this system hinges on a critical challenge: ensuring a durable bond during treatment, while facilitating a controlled mechanical failure upon debonding that preserves the underlying biological substrate. The primary risk lies in the debonding failure occurring at the adhesive–enamel interface, which leads to iatrogenic enamel damage. While the clinical consequences are known, the influence of specific bracket biomechanics—such as the high rigidity of sapphire versus the ductility of metal—on the fracture mechanics at this interface is not fully characterized. This study addresses this engineering gap by investigating the extent of enamel substrate degradation following the debonding of these two distinct materials. We hypothesized that the biomechanical mismatch of rigid sapphire brackets would lead to a more destructive failure mode at the enamel interface compared to metallic brackets and that remineralization would only partially mitigate this surface damage.

## 2. Materials and Methods

### 2.1. Selection and Ethical Approval

This in vitro experimental study was conducted on a sample of 200 intact permanent teeth (incisors, canines, premolars, and molars) extracted for orthodontic and periodontal reasons at the Oral and Maxillofacial Surgery Clinic of Craiova Emergency County Hospital, Romania. Ethical approval for the study was granted by the Commission of Ethics at the University of Medicine and Pharmacy of Craiova (Approval No. 152/11.07.2022), following the principles of the Declaration of Helsinki. Informed consent was obtained from all patients prior to extraction. The inclusion criterion was the use of intact permanent teeth. Teeth that were deciduous or fractured or showed signs of carious or wear lesions were excluded from the study.

### 2.2. Experimental Design and Workflow

**Figure 1:**
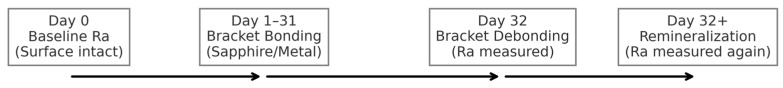
Timeline of enamel surface evaluations: baseline (day 0), bracket bonding (days 1–31), bracket debonding (day 32), and remineralization (day 32+).

The study followed a three-stage experimental design to evaluate enamel surface roughness, as illustrated in the experimental timeline (Figure 1). Measurements were taken in the following stages:

- Stage 1 (Day 0): Baseline assessment of the intact enamel surface.
- Stage 2 (Day 32): Assessment immediately after bracket debonding.
- Stage 3 (Day 32+): Final assessment following a 10-day remineralization protocol.

### 2.3. Sample Size Determination

The sample size was determined a priori using G*Power software (version 3.1) to ensure adequate statistical power [22]. To compare the three measurement stages, the calculation was based on a one-way fixed-effects ANOVA (omnibus test) with the following parameters: a moderate effect size (f = 0.30), a significance level (α) of 0.05, and a desired power (1–β) of 0.80. The G*Power (version 3.1) calculation indicated that a minimum total sample size of 66 teeth (22 per group) was required. Thus, a total of 200 teeth (100 per group) were used to enhance the study’s robustness and exceed this minimum threshold. A post hoc power analysis confirmed that the final sample size yielded a statistical power of 1.0, indicating the study was more than adequately powered to detect significant effects.

### 2.4. Tooth Preparation and Disinfection

Immediately after extraction, teeth were washed with water to remove debris. Subsequently, they were disinfected by immersion in 10% hydrogen peroxide (H_2_O_2_) for 10 min. This protocol was selected based on the literature demonstrating its effectiveness and minimal impact on the enamel structure at the chosen concentration and duration [23]. While higher concentrations or prolonged exposure can alter enamel, the risk was considered clinically negligible under these conditions. Following disinfection, the teeth were stored in deionized water at approximately 23 °C for 24 h to prevent desiccation.

### 2.5. Experimental Group Allocation

Before applying brackets, the teeth were removed from deionized water, dried, and divided into two groups (A and B), with each group containing 100 samples:

- Group A: Teeth with sapphire brackets applied (*n* = 100).
- Group B: Teeth with metallic brackets applied (*n* = 100).

All samples were handled under standardized laboratory conditions to avoid bias in surface analysis.

### 2.6. Bracket Bonding and Photopolymerization

For both experimental groups, a standardized bonding protocol was followed. The buccal enamel surfaces were etched with Transbond XT Etching Gel (St. Paul, MN, USA) for 30 s, rinsed with water for 10 s, and then air-dried. A thin, uniform layer of Transbond™ XT Light Cure (St. Paul, MN, USA) Adhesive was applied and photopolymerized for 10 s using a Demi Plus LED lamp (Kerr Dental, Kloten, Switzerland). Brackets were subsequently bonded using 3M™ Transbond™ XT (Minnesota Mining and Manufacturing Company, St. Paul, MN, USA) adhesive, followed by an additional 10 s of photopolymerization on each side of the bracket to ensure complete curing [22,23]. To minimize variability, all brackets were bonded to the buccal crown surface under constant pressure, and all adhesive procedures were kept consistent across all specimens.

### 2.7. Debracketing Procedure and Post-Treatment Analysis

After a 31-day bonding period, the brackets were removed on day 32. Debonding was performed using a specialized bracket removing plier (Hu-Friedy, Chicago, IL, USA; ORTHO PLIERS, (Hu-Friedy, Chicago, IL, USA) Long Handle, COD 678-220), which applies a squeezing force of approximately 50 N, consistent with standard protocols [23,24]. Immediately following removal, the enamel surfaces underwent a second roughness analysis using both SEM and the TR200 profilometer (Salu Tron GmbH, Frechen, Germany). Subsequently, the remineralization treatment was applied to all specimens, marking the beginning of the final analysis stage.

### 2.8. Remineralization Protocol

Following the post-debonding measurements, a remineralization treatment was applied. The protocol consisted of applying a uniform layer of GC Tooth Mousse^®^ (GC Dental, Luzern, Switzerland) remineralization gel (GC Corporation, Tokyo, Japan), which contains 900 ppm fluoride, to the buccal enamel surface using a sterile microbrush. The gel was left in place for 3 min before being gently rinsed with distilled water, in accordance with the manufacturer’s instructions. This procedure was repeated once daily for 10 consecutive days to simulate a short-term clinical application regimen as recommended for the product.

During this 10-day period, all specimens were stored in artificial saliva (composition: 0.4 g/L NaCl, 0.4 g/L KCl, 0.795 g/L CaCl_2_·2H_2_O, 0.78 g/L NaH_2_PO_4_·2H_2_O, 1 g/L urea, pH 7). This was to provide a stable, ion-rich environment conducive to the remineralization process, thereby better simulating physiological conditions compared to storage in deionized water. The saliva was maintained at 37 °C and refreshed daily to ensure consistent mineral availability.

### 2.9. Instrumentation

The following instruments were used for surface analysis:

- Scanning Electron Microscope (SEM): A Phenom Pure ProX scanning electron microscope (Thermo Fisher Scientific, Waltham, MA, USA), equipped with a backscattered electron detector, was used for qualitative and morphological analysis of the enamel surface. The instrument was operated at an accelerating voltage of 15 keV, and images were captured at magnifications of ×1000 and ×2000.
- A TR200 roughness tester (Salu Tron Messtechnik GmbH, Frechen, Germany) was used for quantitative topographical analysis. The instrument was calibrated to measure the surface roughness parameters Ra (arithmetic mean deviation) and Rz (average height of irregularities) with a precision of ±0.01 μm.

### 2.10. Comparative Roughness Measurement Using TR200 Profilometer

To validate the results of this study, enamel roughness was also measured using the TR200 roughness tester (SaluTron Messtechnik GmbH, Dr.-Gotfried-Cremer-Allee 30/7, Frechen, Germany) (Method 2). During the initial experimental measurements, it was observed that the probe of the roughness tester caused the teeth to overturn, which compromised the stability and measurement accuracy. To address this issue, teeth were stabilized by embedding them in Clarocit Kit acrylic resin (Struers, Pederstrupvej 84 2750, Ballerup, Hovedstaden, Denmark), a material with a polymerization time of just a few minutes at room temperature [25].

**Figure 2:**
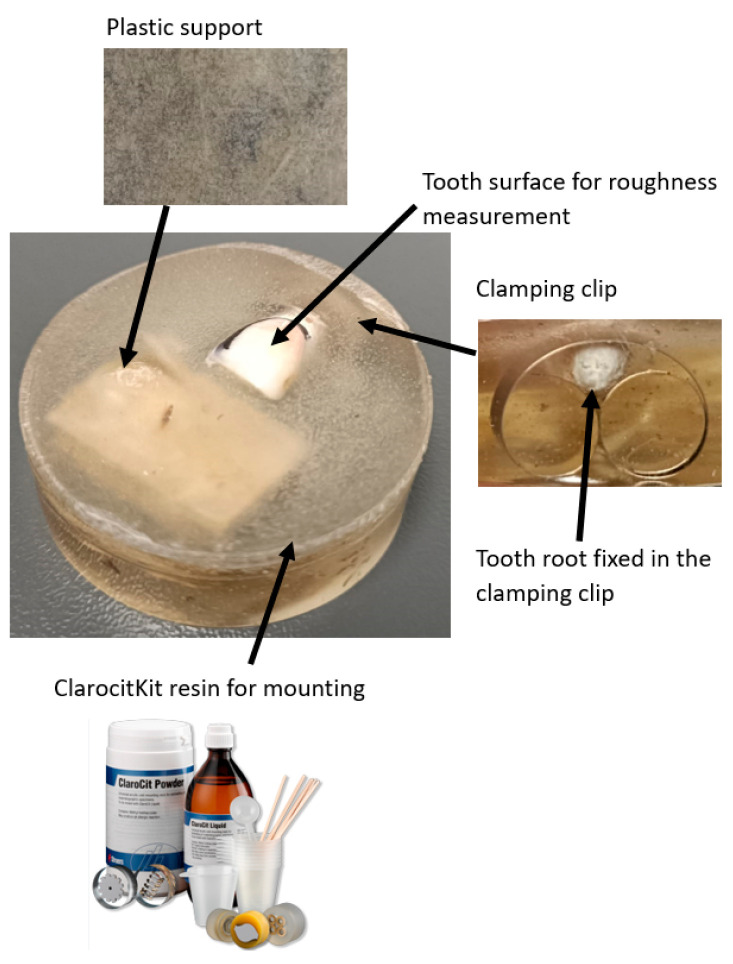
The assembly used to validate the experimental results with the TR200 roughness tester.

**Figure 3:**
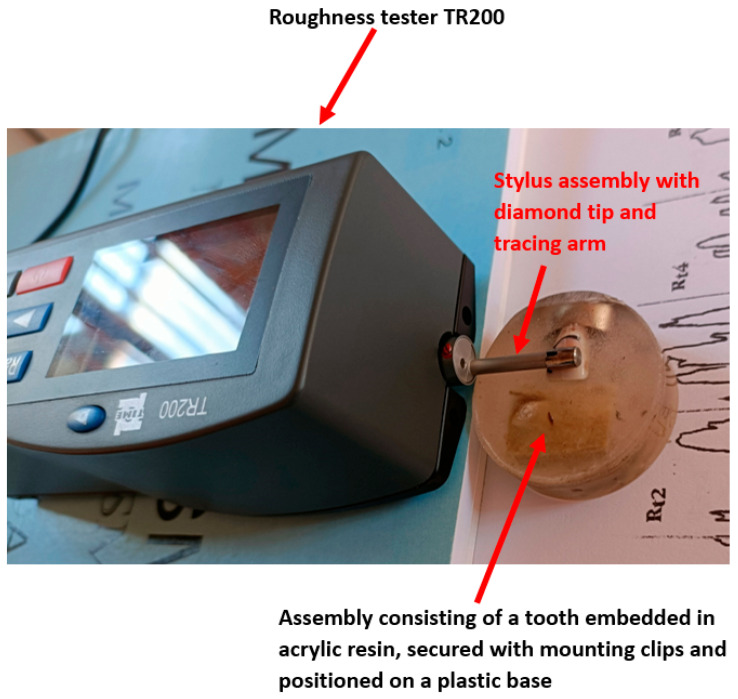
The assembly used during this study with the TR200 roughness tester to validate roughness after the remineralization process.

To ensure stability during TR200 (SaluTron GmbH, Germany) measurements, teeth were embedded in Clarocit acrylic resin using clamping clips to secure positioning, while keeping the enamel surface exposed (Figure 2). This preparation prevented tipping and allowed accurate profilometric readings. Figure 3 illustrates the complete experimental setup with the TR200 (SaluTron GmbH, Germany) device.

During the embedding process, a plastic support was used to prevent the resin from covering the tooth surface, ensuring that only the base was stabilized. Additionally, special clamping clips (Struers, Pederstrupvej 84 2750, Ballerup, Hovedstaden, Denmark) were employed to secure the teeth in place [25]. This approach allowed the teeth to remain stable during roughness measurements without interfering with the enamel surface (see Figure 2).

However, this sample preparation process meant that the comparative TR200 (SaluTron GmbH, Germany) measurements were limited to only those taken after bracket debonding and the application of the remineralization treatment.

### 2.11. Surface Selection and Preparation for SEM Analysis

**Figure 4:**
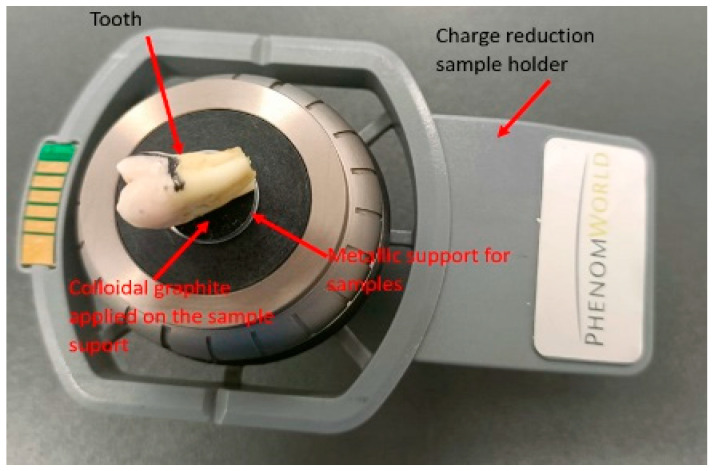
Charge reduction sample holder with tooth and sample support.

The SEM analysis was performed on the buccal enamel surface of intact teeth, specifically in the area where the orthodontic brackets had been bonded. For each sample, the region with the highest visible roughness after debonding was selected for detailed imaging. To prepare the samples for analysis, teeth were mounted on aluminum stubs using double-sided conductive graphite tape to ensure proper adhesion and electrical conductivity. The mounted samples were then placed in a charge reduction sample holder, as shown in Figure 4, and introduced into the SEM chamber.

This technique allows for the analysis of non-conductive samples without the need for metallic sputter coating, thus preserving the original surface topography.

### 2.12. SEM Imaging and 3D Roughness Reconstruction

Using scanning electron (Scientific, Eindhoven, Nederland) microscopy (SEM), morphological micro- and nano-structural images of the enamel surface were captured and saved in BMP format. The images were processed using Phenom Pro Suite (version 2.0) equipped with the 3D Roughness Reconstruction module. This software utilizes ‘shape from shading’ technology to generate three-dimensional images and sub-micrometer roughness measurements. This technology reconstructs the enamel surface topology by combining shading models from four-segment images acquired from the backscatter detector of the electron microscope [26,27,28]. This module provided a three-dimensional representation of the analyzed surface, from which Ra and Rz roughness values were measured linearly. The measurement directions for each analysis were selected directly on the SEM images of the relevant area.

### 2.13. Surface Roughness Analysis

Surface roughness was assessed using parameters Ra (arithmetic average deviation of roughness) and Rz (average height of irregularities), as defined by the SR EN ISO 21920-2:2022 standard [29]. While both parameters were recorded, Ra was selected for subsequent statistical analysis. This choice was based on its widespread use and acceptance in dental material literature as a reliable metric for quantifying overall surface roughness, which ensures the comparability of our results with previous studies.

To minimize observer bias, the operator performing the SEM and TR200 roughness (SaluTron GmbH, Germany) measurements was blinded to the experimental group allocation of each sample.

### 2.14. Statistical Analysis

All data were organized and processed using Microsoft Excel. The statistical analysis was performed using the software GraphPad Prism (version 10.2.0, GraphPad Software, San Diego, CA, USA) and followed a sequential protocol. First, the Grubbs test was applied to identify any potential outliers in the experimental data. A post hoc statistical power analysis was conducted to confirm that the sample size was adequate. The Kolmogorov–Smirnov test, along with Box–Cox transformation for non-normal data, was used to assess the normality of the distribution. Levene’s test was performed to check for homogeneity of variances. Finally, to compare the means between groups, a one-way Analysis of Variance (ANOVA) was used, followed by a post hoc Bonferroni test for pairwise comparisons. A *p*-value of <0.01 was considered statistically significant for all tests.

For the statistical analysis, the obtained data were organized into six groups as follows:

- Group A1: Contains the experimental data for 100 teeth before the bonding of sapphire brackets.
- Group A2: Contains the experimental data for 100 teeth after the debonding of sapphire brackets.
- Group A3: Contains the experimental data for 100 teeth after the debonding of sapphire brackets and the application of the remineralization treatment.
- Group B1: Contains the experimental data for 100 teeth before the bonding of metallic brackets.
- Group B2: Contains the experimental data for 100 teeth after the debonding of metallic brackets.
- Group B3: Contains the experimental data for 100 teeth after the debonding of metallic brackets and the application of the remineralization treatment.

In cases where multiple comparisons were conducted, Bonferroni correction was applied to adjust the significance threshold, thereby minimizing the risk of false-positive results. The corrected alpha level was calculated by dividing 0.05 by the number of pairwise comparisons.

## 3. Results

### 3.1. General Analysis of Enamel Surface Roughness

**Figure 5:**
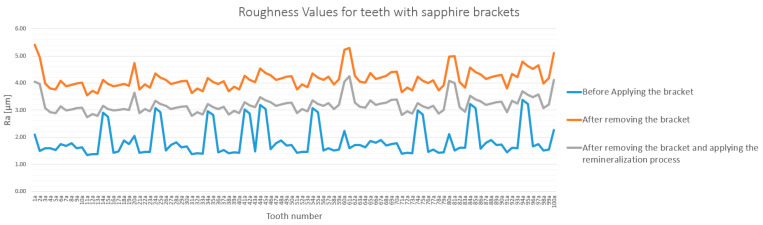
Roughness (Ra) variation for teeth with sapphire brackets in baseline, post-debonding, and post-remineralization stages. The legend entries correspond to the baseline, post-debonding, and post-remineralization stages, respectively.

**Figure 6:**
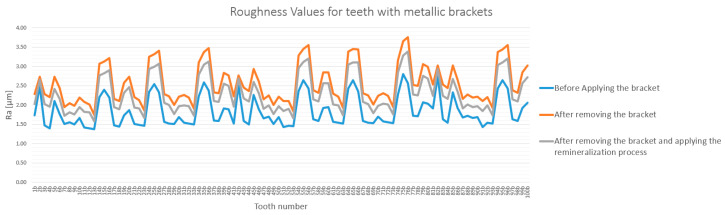
Roughness (Ra) variation for teeth with metallic brackets in baseline, post-debonding, and post-remineralization stages. The legend entries correspond to the baseline, post-debonding, and post-remineralization stages, respectively.

The analysis of enamel surface roughness revealed significant differences both between the experimental groups and across the three measurement stages. The overall trends are presented in Figure 5 and Figure 6.

For the sapphire bracket group (Group A), debonding caused a significant increase in enamel roughness. Ra values rose from a baseline of 1.72–2.05 µm to a post-debonding range of 3.85–4.92 µm. Following the remineralization protocol, roughness values decreased to 3.19–3.78 µm but did not return to baseline levels (Figure 5).

A similar trend was observed in the metallic bracket group (Group B), although the increase was less pronounced. Ra values rose from a baseline of 1.68–1.94 µm to 2.45–2.96 µm after debonding. Following remineralization, roughness decreased to a range of 2.12–2.51 µm, significantly closer to the initial state (Figure 6).

These findings suggest that sapphire brackets cause more considerable and persistent surface damage compared to metallic brackets, and that remineralization is only partially effective in restoring enamel integrity.

### 3.2. Qualitative and Cross-Validation Analysis

**Figure 7:**
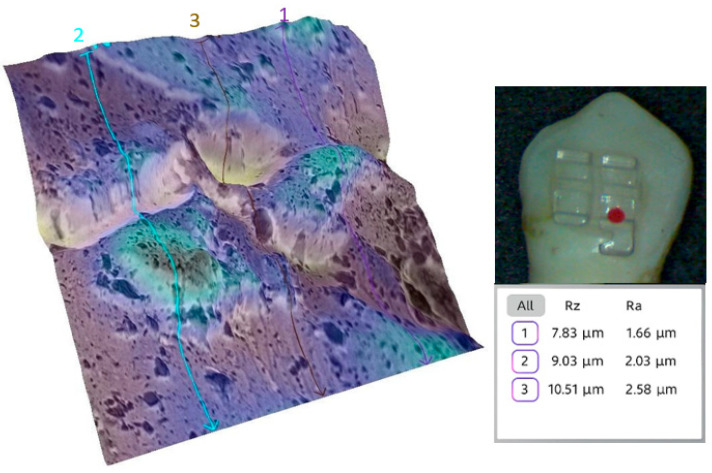
Three-dimensional reconstruction of the enamel of tooth 1a at baseline. The average Ra for the representative area was 2.09 µm.

To illustrate the surface changes at a micro-level, representative teeth from each group were analyzed in detail. Qualitative SEM analysis supported the quantitative findings. For instance, the baseline enamel surface of a representative tooth from the sapphire group (tooth 1a) appeared smooth, with minimal irregularities (Figure 7).

**Figure 8:**
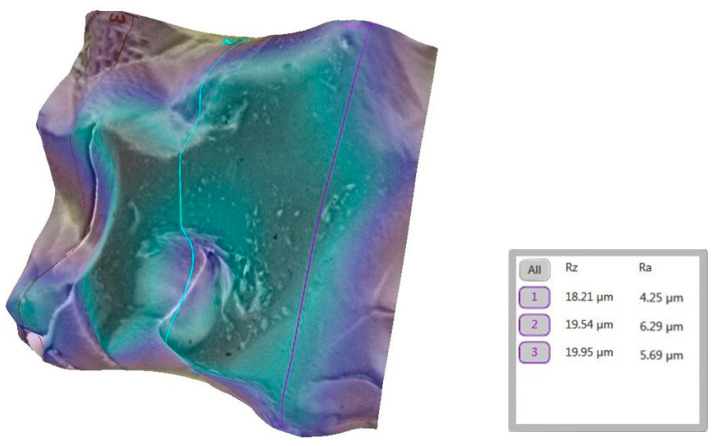
Three-dimensional reconstruction showing the roughness of tooth 1a after removing the sapphire bracket. The average Ra for the representative area increased to 5.41 µm.

A marked increase in roughness was observed after debonding, with pronounced microtopographical changes and higher Ra values (Figure 8).

**Figure 9:**
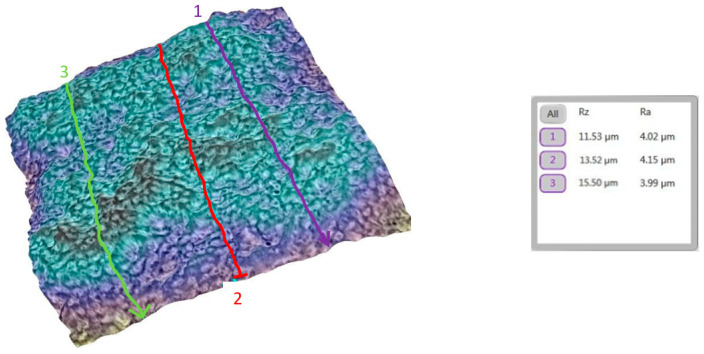
Three-dimensional reconstruction showing the roughness of tooth 1a post remineralization. The average Ra for the representative area was reduced to 4.05 µm.

The remineralization treatment partially smoothed the surface, but irregularities remained visible (Figure 9).

The evolution of the surface profile across the three stages is detailed in Supplementary Figure S1.

A similar analysis was performed for the tooth (metallic bracket).

**Figure 10:**
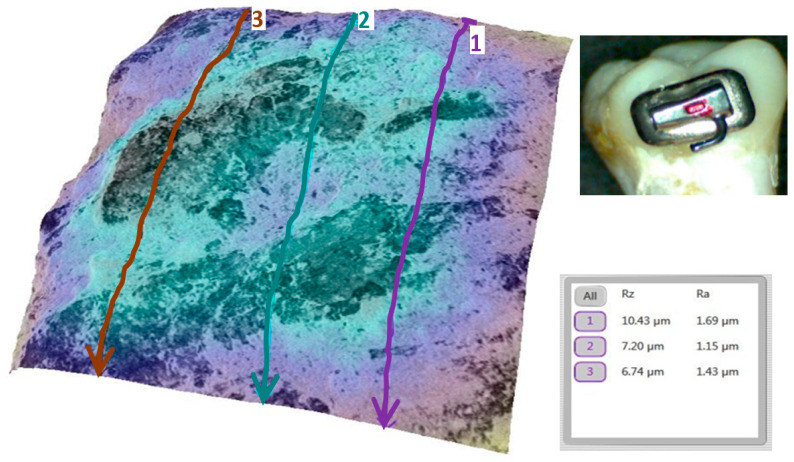
Three-dimensional reconstruction of the analyzed area, showing macroscopic detail with the metallic bracket and the Ra and Rz roughness values for tooth 11b before bonding the bracket. The average Ra for this group was 1.85 µm.

**Figure 11:**
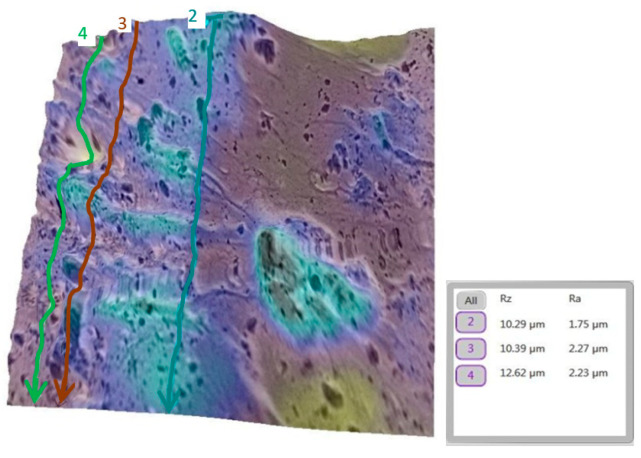
Three-dimensional reconstruction showing the roughness of tooth 11b post debonding (metallic bracket). The average Ra for the representative area increased to 2.08 µm.

**Figure 12:**
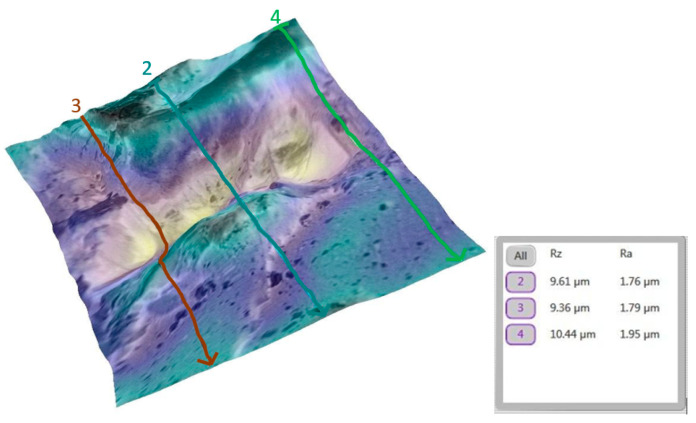
Three-dimensional reconstruction showing the roughness of tooth 11b post remineralization. The average Ra for the representative area was reduced to 1.83 µm.

The progression from a smooth baseline (Figure 10) to a moderately rough post-debonding surface (Figure 11), and a nearly restored post-remineralization surface (Figure 12), is shown.

The profile changes for tooth 11b are summarized in Supplementary Figure S2.

### 3.3. Method Cross-Validation with TR200 Profilometer

**Figure 13:**
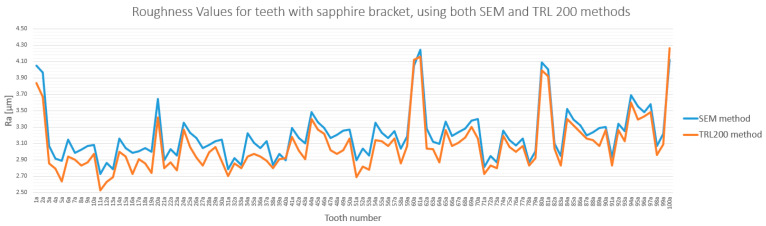
Comparison of roughness values (Ra) obtained via SEM and TR200 profilometer for sapphire bracket group.

**Figure 14:**
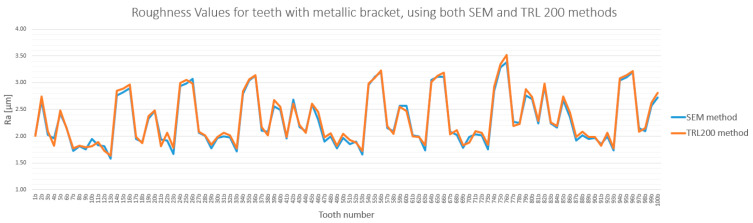
Same as Figure 13 but for metallic bracket group.

The quantitative measurements obtained via SEM were cross-validated using a TR200 roughness( SaluTron GmbH, Germany) tester. The roughness values obtained by both methods were highly consistent, with differences below 7.5% for both the sapphire and metallic bracket groups (Figure 13 and Figure 14). This strong correlation confirms the reliability of the findings and reaffirms that enamel roughness is significantly higher on teeth treated with sapphire brackets.

### 3.4. Detailed Statistical Confirmation

A detailed statistical analysis was performed to confirm the significance of the observed differences. Preliminary analysis using the Grubbs test (α = 0.01) showed no outliers in any dataset. A post hoc power analysis confirmed the sample size was adequate to detect significant effects (power = 1.0). The statistical power for all groups was calculated to be 1.0, confirming that the sample size of 100 teeth per group was adequate (see Supplementary Table S1 for full details regarding the results).

Data normality was tested using the Kolmogorov–Smirnov test. For groups A1 and B1, which did not follow a normal distribution (*p* < 0.01), a Box–Cox transformation was applied prior to analysis. Full results of the normality tests are available in Supplementary Tables S2 and S3. Levene’s test confirmed the homogeneity of variances across all groups (*p* > 0.01) (see Supplementary Table S4).

One-way ANOVA tests revealed statistically significant differences within both the sapphire groups (A1, A2, A3) (F = 700.34, *p* < 0.001) and the metallic groups (B1, B2, B3) (F = 59.87, *p* < 0.001). Furthermore, direct comparisons showed significant differences between the sapphire and metallic groups in the post-debonding (A2 vs. B2: F = 627.37, *p* < 0.001) and post-remineralization (A3 vs. B3: F = 254.56, *p* < 0.001) stages.

Post hoc pairwise comparisons using the Bonferroni correction (adjusted α = 0.0033) confirmed that nearly all differences between stages and bracket types were statistically significant (Table 1). The 95% confidence intervals also reflected these differences, with narrower intervals indicating higher measurement precision in post-treatment groups (Table 2).

In conclusion, the statistical data confirmed the following:

1. Both bracket types significantly increased enamel roughness.
2. Sapphire brackets caused a statistically significant higher increase in roughness than metallic brackets did.
3. Remineralization significantly reduced roughness in both groups, but failed to restore the enamel to its original state.

## 4. Discussion

Remineralization significantly reduced enamel roughness in both groups, though the values did not return to baseline (*p* < 0.001). In the sapphire group (A), Ra decreased from 4.14 ± 0.36 µm after debonding to 3.19 ± 0.30 µm post remineralization, corresponding to a 23% reduction. In the metallic group (B), Ra decreased from 2.56 ± 0.52 to 2.29 ± 0.48 µm, a 10% reduction. Despite these improvements, both groups retained significantly higher Ra values compared to baseline (A1 = 1.87 ± 0.57 µm; B1 = 1.85 ± 0.40 µm), confirming that remineralization only partially restored enamel smoothness.

The findings of this study indicate that bracket material has a significant effect on enamel surface roughness following debonding. Notably, sapphire brackets were associated with a greater increase in roughness compared to metallic brackets. This effect may result in a higher hardness and rigidity of sapphire, which may cause more microstructural damage to the enamel during bonding and removal. These results are consistent with previous studies reporting increased roughness after debonding ceramic or sapphire brackets, which exhibit a higher modulus of elasticity and surface hardness [30,31,32].

By contrast, metallic brackets appear to exert a less aggressive effect on the enamel surface, potentially due to their greater flexibility and improved adaptation during bonding, resulting in lower post-debonding roughness values [25,32,33].

From a biomechanical standpoint, the increased enamel damage associated with sapphire brackets can be attributed to the significant mismatch in the modulus of elasticity at the material interface and the principles of fracture mechanics. Monocrystalline sapphire possesses an extremely high modulus of elasticity, making it exceptionally rigid. During debonding, this rigidity prevents the bracket from deforming and absorbing energy, causing a high concentration of stress to be transferred directly to the enamel-adhesive interface. As enamel is a brittle, ceramic-like material, this concentrated stress is highly effective at initiating and propagating microcracks at the surface, leading to a cohesive fracture within the enamel itself and a significant loss of substance. By contrast, metallic brackets, while also stiff, are more ductile. They can undergo slight plastic deformation under the debonding force, which dissipates some of the applied energy and distributes the stress more broadly. This mechanism reduces the peak stress exerted on the enamel, making it more likely for the failure to occur in a benign fashion within the adhesive layer or at the bracket–adhesive interface, thus preserving the enamel surface [15]. Therefore, the greater surface roughness is not merely a result of hardness, but a direct consequence of a more destructive, brittle failure mode induced by the rigid biomechanical properties of sapphire.

Statistical analysis confirmed significant differences in enamel roughness across the three experimental stages. The methodological strength of this study was significantly enhanced by a dual-assessment approach, using both scanning electron (Scientific, Eindhoven, Nederland) microscopy (SEM) and a TR200 roughness( SaluTron GmbH, Germany) tester for cross-validation. As detailed further in the methodological considerations, the high consistency between the two methods (<7.5% difference) underscores the reliability and accuracy of our findings [26,34].

### 4.1. Comparisons with Other Studies

Several previous studies have investigated how bracket material affects enamel surface roughness following orthodontic treatment, consistently reporting increased roughness associated with ceramic-based brackets compared to metallic ones. Vidor et al. [35] observed roughness increases of 30–50% with ceramic brackets, particularly when traditional adhesive removal methods were used. This aligns with our findings, where sapphire brackets—classified as monocrystalline ceramics—produced even higher roughness values, ranging from 3.85 to 4.92 µm, representing up to a 65% increase from baseline.

Similarly, Thawaba et al. [36] emphasized that high-hardness materials such as sapphire increase the likelihood of microcrack formation during debonding, due to their rigidity and high modulus of elasticity. Our study corroborates this, showing significantly greater enamel alteration after the removal of sapphire brackets in contrast to metallic ones, under standardized conditions.

Kakaboura et al. [37] also reported that metal brackets, due to their mechanical adaptability and lower surface hardness, result in lower enamel roughness values post debonding. In our study, the roughness associated with metallic brackets ranged between 2.45 and 2.96 µm—significantly lower than the values observed in the sapphire group.

In line with our results, Karan et al. [38] found that ceramic brackets can lead to roughness values between 2.5 and 4.0 µm. The slightly higher range in our study may reflect the unique properties of monocrystalline sapphire, which may lead to a stress concentration greater than those of polycrystalline ceramics during bracket removal.

Other studies further support this trend. For instance, [39] reported post-debonding roughness values of 2.8–4.5 µm for ceramic brackets, while [40] found values ranging from 2.0 to 3.0 µm for metallic brackets—comparable to our findings in the metal group. These consistent data across multiple studies reinforce the enamel-preserving advantages of metallic brackets.

In summary, the comparative analysis confirms that bracket material plays a critical role under the post-debonding enamel surface condition. Rigid, high-hardness brackets such as sapphire pose a greater risk to enamel integrity. Unlike many previous investigations, this study additionally explored the effect of remineralization protocols on post-debonding roughness, contributing new evidence with both clinical and scientific relevance. The validity of these findings and their comparison to the literature are fundamentally grounded in the rigorous design of this study, whose methodological aspects are detailed below.

### 4.2. Methodological Considerations

The study employed both scanning electron (Scientific, Eindhoven, Nederland) microscopy (SEM) and a TR200 roughness (SaluTron GmbH, Germany) tester to assess enamel surface roughness, enabling comprehensive and cross-validated measurements. The consistency between the two methods, despite their distinct operational principles, supports the reliability of the results. While the TR200 is typically suited for larger surface areas, careful sample stabilization—achieved by embedding the teeth in acrylic resin—allowed accurate measurements on limited enamel surfaces [34].

SEM offered high-resolution visualization but required meticulous sample preparation to avoid imaging artifacts [26]. Conversely, TR200 (SaluTron GmbH, Germany) measurements on small, curved surfaces may be more prone to variability, reinforcing the need for precise calibration and standardized positioning [34].

Another methodological aspect relates to the inclusion of different tooth types (incisors, canines, premolars, and molars). This choice was made to increase the clinical relevance and generalizability of our findings, as orthodontic treatment involves the entire dental arch. However, we acknowledge that these teeth vary significantly in enamel thickness and surface anatomy, which is a potential confounding factor. While bracket bonding was standardized to the buccal crown surface and teeth were randomly distributed to mitigate this variable, we recognize that randomization alone may not fully eliminate its influence. A dedicated subgroup analysis (e.g., comparing incisors to molars) was beyond the scope of this study but represents a valuable direction for future research to isolate tooth-specific effects.

Although the study was conducted in an orthodontic context, its findings have broader implications. Enamel surface quality influences outcomes across multiple dental specialties, including restorative, preventive, and adhesive dentistry. The insights gained here may inform more effective protocols for bonding, polishing, and material selection.

Our results confirm the partial efficacy of GC Tooth Mousse^®^ (GC Dental, Luzern, Switzerland) in reducing enamel surface roughness after bracket debonding. The quantitative analysis revealed a greater relative reduction in roughness in the sapphire group (23%) compared to the metallic group (10%). We interpret this not as an enhanced efficacy of the treatment on more damaged surfaces, but rather as a reflection of the higher initial damage caused by the sapphire brackets. Despite these statistically significant reductions, roughness in both groups did not return to baseline values, suggesting that the structural alterations to the enamel persist despite the remineralizing intervention.

These findings are consistent with previous reports which also indicate a limited but measurable recovery of enamel topography following the application of remineralizing agents such as those containing CPP-ACP [30,38,39]. Crucially, the final Ra values in our study remained well above the clinical threshold of 0.2 µm associated with significant plaque retention. This highlights a key limitation of current remineralization strategies: while they can mitigate some irregularities, they do not fully reverse the damage to a clinically ideal state. Therefore, they should be considered a supportive measure rather than a complete restorative solution.

### 4.3. Clinical Implications

The significantly higher enamel roughness observed after debonding sapphire brackets raises important clinical concerns. Increased surface irregularities have been linked to enhanced plaque retention, bacterial colonization, and a greater risk of caries and enamel degradation. These risks might be more pronounced in patients with compromised oral hygiene, prolonged orthodontic treatment, or pre-existing demineralization, although clinical confirmation is required. Therefore, bracket material selection should be guided not only by esthetic considerations, but also by its potential effect on enamel surface integrity and long-term oral health [41,42,43,44,45].

This study suggests that post-debonding remineralization protocols might offer some benefit in reducing enamel roughness. The application of a fluoride-containing agent (GC Tooth Mousse^®^) resulted in a measurable reduction in surface roughness, although enamel was not fully restored to its baseline condition. These findings suggest that remineralization should be considered a supportive strategy rather than a definitive restorative intervention. Our findings on the partial efficacy of GC Tooth Mousse^®^ (GC Dental, Luzern, Switzerland) highlight the limitations of current supportive therapies. While this product, containing fluoride and Recaldent™ (GC Dental, Luzern, Switzerland) (CPP-ACP), offers measurable benefits, its inability to fully restore the enamel surface underscores the need for more advanced strategies. The field of remineralization is rapidly evolving, with promising results from agents like bioactive glass, which releases calcium and phosphate ions to form a hydroxyapatite-like layer, and nanohydroxyapatite particles, which can directly fill enamel microporosities. Comparative studies are essential to determine whether these alternative agents offer superior outcomes in restoring enamel integrity post debonding. Further research should therefore focus not only on optimizing application protocols but also on comparing the long-term efficacy of these next-generation materials [43,46].

Debonding and adhesive removal techniques are also critical in determining the extent of enamel damage. Conventional mechanical methods may result in microfractures and surface loss. Alternative approaches, including laser-assisted debonding, air-abrasion systems, or enzymatic adhesives, may offer less invasive options with reduced enamel effect [46,47,48,49,50,51,52,53,54,55,56,57].

In addition, long-term clinical studies are essential to assess the persistence and clinical relevance of post-treatment surface roughness. Most current studies, including the present one, evaluate roughness immediately after debonding, leaving the longitudinal effects on caries susceptibility, patient comfort, and esthetic outcomes relatively unexplored. Investigating how enamel roughness evolves over time could lead to evidence-based preventive strategies [45,58,59,60].

Although the observed differences in surface roughness were statistically significant, it is important to assess their clinical relevance. Roughness influences plaque retention, bacterial adhesion, and enamel demineralization. According to Pratten and Wilson (1999), even minor increases in roughness can promote microbial colonization, especially in difficult-to-clean areas [61]. Aljamhan et al. (2021) further emphasized that changes in enamel texture may affect post-treatment esthetics and susceptibility to demineralization [62]. From a biomechanical perspective, Wang et al. (2020) [63]. demonstrated that surface characteristics—including roughness—can impact force delivery and patient comfort. Thus, the differences observed in this study, though moderate in magnitude, may have practical consequences during long-term aligner or bracket wear and warrant further clinical investigation

Enamel preservation during orthodontic treatment is a multifactorial challenge involving bracket design, bonding and etching protocols, adhesive composition, removal techniques, and post-treatment care. Each of these elements may influence the degree of enamel alteration, and their combined effects warrant further investigation. Integrating preventive strategies, including informed material selection and remineralization therapy, may help reduce iatrogenic effects and might contribute to improved enamel preservation, pending further clinical validation.

The study also highlights a clinical trade-off: while sapphire brackets offer esthetic benefits, they tend to compromise enamel surface integrity more than metal brackets. This insight is relevant not only for orthodontic specialists but also for restorative dentists and researchers in dental biomaterials.

In addition to bracket type, other factors such as the type of bonding agent, etching protocol, and adhesive removal technique can also influence enamel surface integrity. The mechanical properties of the adhesive, its interaction with enamel prisms, and the technique used for clean-up may collectively contribute to post-treatment roughness. Future studies should consider isolating these variables to better understand their individual and combined effects.

Given the in vitro nature of the current study, these implications should be interpreted with caution and should not be directly extrapolated to clinical settings without further in vivo validation.

### 4.4. Strengths and Contributions of Study

This study offers several noteworthy strengths. First, it provides a direct and controlled comparison between sapphire and metallic brackets using a well-standardized in vitro design and a substantial sample size (*n* = 200), thereby enhancing the statistical power and reliability of the findings. Second, it is among the few investigations to evaluate enamel surface roughness not only after bracket debonding but also following remineralization, offering a more comprehensive view of post-treatment enamel recovery.

Furthermore, the methodological rigor was reinforced by the use of two complementary assessment techniques (SEM and TR200). As previously discussed, this dual-modality approach ensured both high-resolution morphological evaluation and accurate, reproducible quantitative measurements.

Beyond methodological strengths, the study provides clinically relevant insights. Its findings are applicable to both orthodontic and restorative dentistry, as enamel preservation is a shared priority. The data presented here may inform clinical decision making regarding bracket selection, bonding and removal protocols, and the use of adjunctive remineralization strategies.

Overall, the study contributes meaningful evidence to the literature and helps bridge the gap between technical orthodontic procedures and biological outcomes related to enamel integrity.

### 4.5. Study Significance and Scope

While this in vitro study cannot fully replicate intraoral conditions, it was designed with methodological rigor and statistical validation to yield reproducible results. This study employed an in vitro model to evaluate enamel surface roughness associated with different bracket materials and post-debonding remineralization. Standardized protocols, a controlled experimental design, and the use of both SEM and TR200 (SaluTron GmbH, Germany) instruments contributed to the internal validity of the measurements. However, as acknowledged in the limitations section, in vitro models cannot fully replicate intraoral conditions, and the results should be interpreted within this context. While the findings may serve as a useful preliminary reference, further in vivo studies are needed to validate these outcomes and assess their clinical applicability.

These results should be interpreted cautiously and confirmed by further in vivo studies before any clinical recommendations are made.

### 4.6. Clinical Significance of Surface Roughness Changes

We recognize the importance of distinguishing statistical significance from clinical relevance in enamel surface roughness. It is widely accepted that Ra values above approximately 0.2 μm are associated with enhanced plaque retention and increased risk of caries development. In our study, even the lowest Ra measurements—observed in the sapphire plus remineralization group (mean ≈ 3.19 µm)—remain well above this clinical threshold. The metallic bracket groups showed slightly lower Ra values (≈2.3 µm), but still far above the 0.2 µm plaque-retention threshold. These findings indicate that, although remineralization reduces surface roughness, it does not restore enamel to a state considered clinically safe for plaque accumulation.

Moreover, a recent in vivo study demonstrated that enamel roughness values of 0.2 μm or higher significantly correlate with increased bacterial adhesion in oral biofilms. This underscores that even sub-micron differences in Ra may have meaningful biological implications. Altogether, our results suggest that current protocols for bracket removal and remineralization, while effective in smoothing enamel surfaces, may require further optimization to approach clinically acceptable roughness levels. Clinical studies are warranted to confirm whether these residual roughness values materially affect long-term oral health outcomes following orthodontic debonding [44,63].

### 4.7. Study Limitations

This in vitro study has inherent limitations. While laboratory conditions ensure control and repeatability, they fail to replicate intraoral complexities such as saliva, pH changes, biofilm, occlusal forces, diet, and hygiene habits. Specifically, the use of a standardized artificial saliva, while an improvement over an inert medium, cannot fully mimic the dynamic buffering capacity and complex organic composition of natural saliva. Additionally, extracted teeth, though practical, cannot fully reflect individual differences in enamel structure and biological response.

While surface roughness is a valid indicator of enamel integrity, it captures only one aspect of enamel health. The short duration of the remineralization protocol also limits its relevance to long-term outcomes, warranting caution in clinical interpretation.

The controlled in vitro design minimized confounding factors, strengthening internal validity and enabling reliable material comparisons. The results offer a reproducible basis for future clinical research on enamel preservation post debonding.

## 5. Conclusions

The debonding of both sapphire and metallic brackets caused a statistically significant increase in enamel surface roughness, with sapphire brackets inducing significantly more surface damage than metallic ones.

While a 10-day remineralization protocol with GC Tooth Mousse^®^ resulted in a statistically significant reduction in roughness, this improvement was only partial.

Crucially, the final Ra values in both groups (2–4 µm) remain far above the clinically accepted threshold of 0.2 µm for plaque retention. Therefore, the main conclusion of this study is that, under these in vitro conditions, the tested protocol leaves the enamel surface in a highly unfavorable clinical state, posing a significant risk for plaque accumulation and caries. Remineralization should be viewed as a minor palliative measure rather than a restorative solution.

These findings highlight a clinical trade-off between the esthetics of sapphire brackets and the superior enamel preservation offered by metallic brackets. There is an urgent need to develop less invasive debonding techniques and more effective surface restoration strategies, such as those involving bioactive materials, to better preserve enamel integrity in orthodontic practice.

## Figures and Tables

**Table 1:** Post hoc pairwise comparisons (Bonferroni) of the most relevant mean differences for Ra (µm).

Comparison	Description	Mean Difference (µm)	Statistically Significant (*p* < 0.0033)
A1 vs. A2	Sapphire: Baseline vs. Post-debonding	2.278	Yes
A2 vs. A3	Sapphire: Post-debonding vs. Post-remineralization	0.950	Yes
A2 vs. B2	Sapphire vs. Metallic (Post-debonding)	1.585	Yes
A3 vs. B3	Sapphire vs. Metallic (Post-remineralization)	0.898	Yes
B1 vs. B2	Metallic: Baseline vs. Post-debonding	0.713	Yes
B2 vs. B3	Metallic: Post-debonding vs. Post-remineralization	0.262	Yes

**Table 2:** 95% confidence intervals (CIs) for Ra values (µm) across experimental groups.

Group	Mean Ra (µm)	SD	n	95% CI Lower	95% CI Upper
A1	1.8665	0.5747	100	1.754	1.979
A2	4.1441	0.3622	100	4.073	4.215
A3	3.1946	0.2971	100	3.136	3.253
B1	1.8453	0.3977	100	1.768	1.922
B2	2.5586	0.5161	100	2.457	2.660
B3	2.2962	0.4771	100	2.203	2.390

## Data Availability

The original contributions presented in this study are included in the article and Supplementary Material. Further inquiries can be directed to the corresponding authors.

## Supplementary Materials

The following supporting information can be downloaded at: https://www.mdpi.com/article/10.3390/bioengineering12101041/s1. Supplementary Tables S1–S4 are available and include power analysis, normality assessments, and variance homogeneity tests supporting the statistical analysis. Supplementary Figures S1 and S2, which show the surface variation of representative teeth, were moved to the Supplementary Materials in order to avoid redundancy with the roughness values already presented in the main text.
