# Supplementary material for: Orthodontic Bracket Removal and Enamel Roughness: Comparing the Effects of Sapphire and Metallic Brackets in an In Vitro Study
Source: Bioengineering (Basel). 2025 Sep 28;12(10):1041. doi: 10.3390/bioengineering12101041 (PMC12561668; doi:10.3390/bioengineering12101041)
Supplement: Supplementary file 1 [file bioengineering-12-01041-s001.zip › bioengineering-3883688 - Supplementary Material.pdf]

Supplementary Material – Statistical Assumptions for Surface Roughness (Ra) Analysis

This supplementary document provides detailed information on the statistical assumptions tested prior to conducting parametric analyses in the study.

1. Normality Assessment

The normality of the data distribution was assessed using the Kolmogorov-Smirnov test. For baseline groups A1 and B1, which did not initially follow a normal distribution ($p < 0.01$), a Box-Cox transformation was applied. After transformation, all groups were confirmed to follow a normal distribution ($p > 0.01$), as detailed in Supplementary Table S1.

2. Homogeneity of Variances

Levene's test indicated homogeneity of variances across all groups ($p > 0.01$), as shown in Supplementary Table S2. These results justify the use of one-way ANOVA followed by Bonferroni post-hoc comparisons.

By comparing A2 and B2 the next results were obtained:

- ANOVA test statistic: 627.378
- p-value: $2.711 \cdot 10^{-63}$.

By comparing A3 and B3 the next results were obtained:

- ANOVA test statistic: 254.568
- p-value: 2.155·10-37.

P-value is lower than 0.01 conforming that there were significant differences between the results obtained for all the compared groups.

Table S1. p-values obtained with Bonferroni correction for A1, A2, A3 groups.

Group	Mean Difference	p-value	α
A1 vs A2	2.2833	$2.28 \cdot 10^{-16}$	0.0033
A1 vs A3	1.3334	$1.33 \cdot 10^{-16}$	
A2 vs A3	-0.9499	$9.5 \cdot 10^{-13}$	

Table S2. p-values obtained with Bonferroni correction for B1, B2, B3 groups.

Group	Mean Difference	p-value	α
B1 vs B2	0.7133	$1.25 \cdot 10^{-16}$	0.0033
B1 vs B3	0.4509	$3.42 \cdot 10^{-15}$	
B2 vs B3	-0.2624	$2.75 \cdot 10^{-4}$	

Table S3. Statistical power analysis for all experimental groups.

Group	Mean	Standard Deviation	Sample Size	Effect Size	Statistical PowerA1.
A1	1.8665	0.5747	100	3.2477	0.01:1.0
A2	4.1441	0.3622	100	11.4423	0.01:1.0
A3	3.1946	0.2971	100	10.7536	0.01:1.0
B1	1.8453	0.3977	100	4.6402	0.01:1.0
B2	2.5586	0.5161	100	4.9573	0.01:1.0
B3	2.2962	0.4771	100	4.8125	0.01:1.0

Table S4. Results of the Kolmogorov-Smirnov (KS) test for data distribution.

Group	Kolmogorov-Smirnov Statistic	p-value
A1	0.2652	$1.07 \cdot 10^{-6}$
A2	0.1101	0.16389
A3	0.12761	0.07
B1	0.1744	0.00396
B2	0.1583	0.01177
B3	0.1532	0.0163

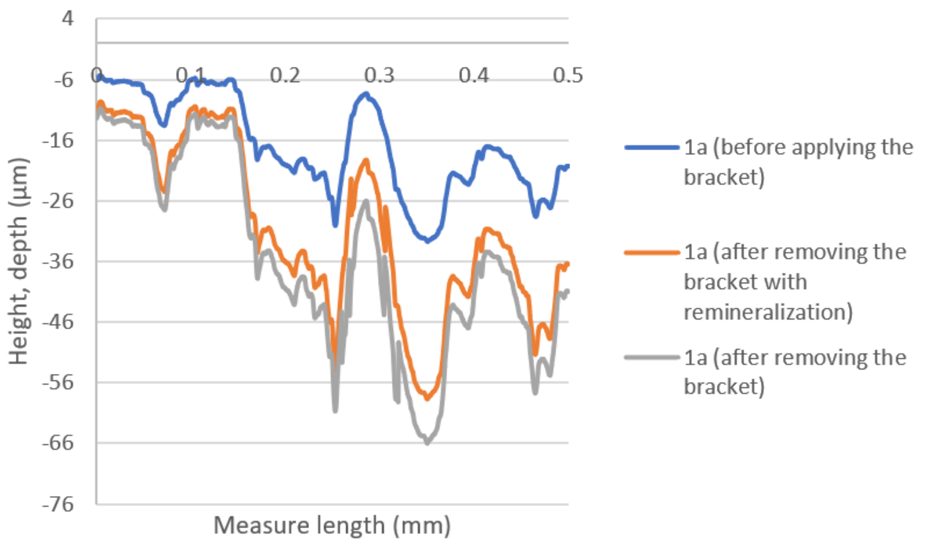

Figure S1. Variation in the surface of tooth 1a (anterior area).

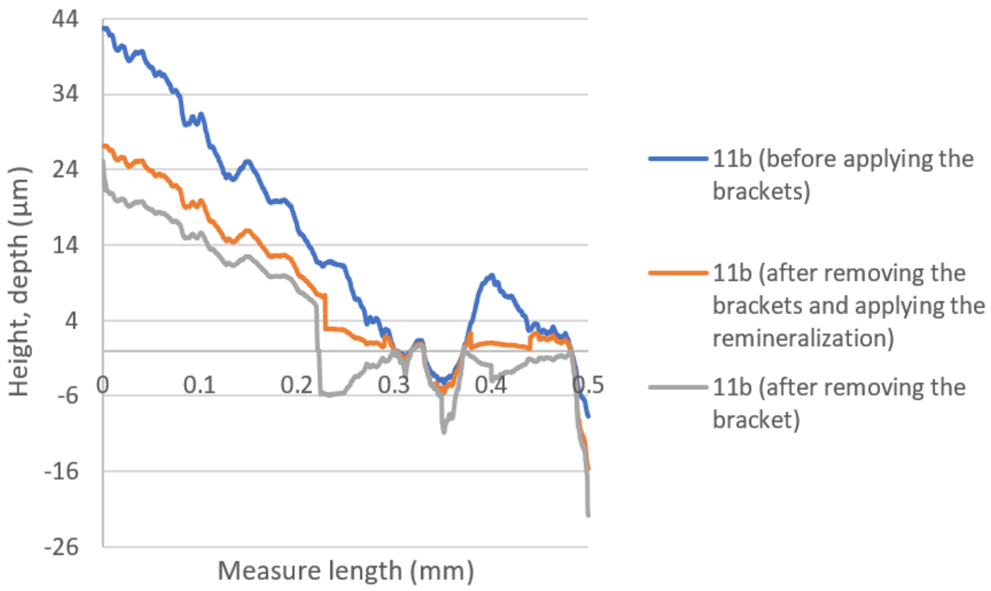

Figure S2. Variation in the surface of tooth.
